# Epidemiology of group A rotavirus in children under five years of age with gastroenteritis in N’Djamena, Chad

**DOI:** 10.1186/s12879-023-08647-5

**Published:** 2024-01-22

**Authors:** Bertrand Djikoloum, Mahamat Fayiz Abakar, Valentine Ngum Ndze, Rahinatou Ghapoutsa Nkandi, Carine Ngah Enjeh, Pidou Kimala, Jean Paul Assam Assam, Maurice BODA

**Affiliations:** 1 Department of Microbiology, Faculty of Science, University of Yaounde I, Yaoundé, Cameroon; 2 Institut de Recherche en Elevage pour le Développement (IRED), N’Djaména, Chad; 3 Faculty of health Sciences, University of Buea, Buea, Cameroon

**Keywords:** Rotavirus, Epidemiology, Gastroenteritis, N’Djamena

## Abstract

**Background:**

Group A Rotaviruses (RVA) is one of the most common causes of severe diarrhoea in infants and children under 5 years of age. Unlike many countries in the world where RVA surveillance/control is active, in Chad , there is currently no applied RVA immunization program and surveillance strategy. The present study aims to determine the prevalence and associated risk factors of RVA gastroenteritis among children under five years of age in N’Djamena.

**Method:**

This study comprised two parts: (1) A cross-sectional study carried in four hospitals in N’Djamena between August and November 2019, to determine infection risk factors and evidence of RVA infection among children aged five and below, consulted or hospitalized for diarrhea. An ELISA based RVA VP6 protein detection was used to determine RVA infection prevalence. Infection results and sociodemographic data were statistically analysed to determine RVA infection risk factors. (2) A retrospective study that consisted of analysing the records of stool examinations of the period from January 2016 to December 2018, to determine the prevalence of infectious gastroenteritis among the target population.

**Results:**

For the cross-sectional study, RVA infection prevalence was 12.76% (18/141) with males (61.11%) being more affected (sex ratio: 1.57). Children below 12 months were the most affected age group (44.44%) and 44.4% were malnourished. The mean Vesikari score shows that 38.8% of children have a high severity level and 41.1% have a moderate level. For the retrospective study, 2,592 cases of gastroenteritis hospitalization were analysed; 980 out of 2,592 cases (37.81%) of hospitalization due to diarrhoea were due to diarrhoeagenic pathogens including *Emtamoeba hystolitica, Gardia lamblia, Trichomonas hominis, Hymenolepis nana, Escherichia coli, Shigella spp, Proteus mirabilis*, and *Klebsiella oxytoca*. Cases of diarrhoea with negative pathogen search were 1,612 cases (62.19%). The diarrhoea peak was observed during the dry seasons, and the age group under 11 months was the most affected was (57.3%).

**Conclusion:**

This study describes the evidence of RVA infection among diarrhoeic children below five years of age in N’Djamena, thus indicates a serious health burden. Malnourishment younger age was the higher risk factor. Further studies are needed to determine the circulating strains prior to considering introduction of RVA vaccine and setup a routine rotavirus surveillance in Chad.

## Introduction

Rotaviruses are member of the *Reoviridae* family with a size of 70–75 nm and are classified into 10 serogroups (A-J) based on the structural intermediate capsid protein VP6. Humans are more susceptible to rotavirus group A (RVA), which represent more than 90% among non-immunocompromised humans. RVA also affect domesticated animals, and is thus recognized as a zoonotic pathogen [1]. Two structural outer capsid proteins, VP7 (G glycoprotein) and VP4 (P glycoprotein) respectively, define the G and P genotypes of the virus. These major antigens elicit the production of neutralising antibodies in the host and are thought to be important for vaccine development. These antigens allow the classification of rotaviruses into a dual nomenclature system, depending on the G–P antigen combination [2].

Infectious gastroenteritis are common human diseases with high morbidity and often high mortality among children under 5 years old. Diarrheagenic bacteria and group A rotavirus (RVA) as well as Norovirus are the most prevalent and widely distributed causes of these diseases worldwide. Rotavirus (RV) accounts for 258 million of cases globally, with 128,500 death yearly [3]. RV is considered to be the third pathogen associated with mortality in children, behind malaria (5,17,000 deaths) and pneumonia (3,59,000 deaths) [4]. RVA is responsible for 34% of acute and severe diarrhoea cases in children under five years of age in countries without RVA vaccine [5].

Vaccination remains the main counter measure currently recommended by the World Health Organization to reduce the disease burden [6] [7]. Studies have shown that vaccination, significantly reduced RVA diarrhoea cases among children under 5 years old [8, 9]. Early complimentary feeding, nutritional status, dehydration and low age (less than 2 years) are important risk factors associated with rotavirus diarrhoea [10, 11].

In Africa, rotavirus is an important cause of severe diarrheal disease in children 5 years of age [12]. Because of its high burden, more than 34 countries in Africa have introduced rotavirus vaccine to their national immunization programs, adopting the monovalent Rotarix, Rotavac, and Rotasiilor the pentavalent RotaTeq [13, 14]. Prior to vaccine introduction, RVA infection prevalence was estimated at 42% in resource limited African countries. With the introduction of RVA vaccine in many African countries, the prevalence dropped to an estimate of 21% [14]. In 2016 RVA prevalence in Sub-Saharan Africa was, with a recorded death cases of 104,733 out of 128 500 deaths cases obtained globally [3]. The circulating G and P rotavirus strains are highly diverse; the most common G types detected are G1, G2, G3 and G9, and the most common P types are P [8], P [6] and P [4]. There is also a high proportion of unusual P/G combinations detected, suggesting viral reassortment, and evidence of zoonotic rotavirus transmission, as confirmed with emergence and spread across Africa of serotype G9 and a high prevalence of the P [6] VP4 genotype [15].

In Chad, 30 to 39% of deaths from diarrhoea in children under five could be attributable to RV [3] and the prevalence was reported around 48% [16]. Despite this diarrhoea related high mortality among children, RV vaccine has not yet been introduced in Chad, and only very few hospitals include RV among the diagnosis panel of gastroenteritis in children. Therefore, this study addresses the question of RVA implication and impact in children health in Chad Republic with main aim to determine infection prevalence and associated risk factors of RVA infection in children under five years of age in N’Djamena.

## Materials and methods

### Type of study, study population and samples collection

This study consisted of two parts: a cross-sectional part and retrospective part.

The cross-sectional study conducted between August and November 2019, was carried out in 4 hospitals in N’Djamena: “*Centre Hospitalier Universitaire de la Mère et de l’Enfant*” (CHUME), “*Complexe Hospitalo-Universitaire le Bon Samaritain*” (CHUBS), “*Hôpital de l’Amitié Tchad-Chine*” (HATC) and “*Hôpital Notre Dame des Apôtres*” (HNDA). The study population were children under 5 years of age hospitalized or seen for acute non-bloody gastroenteritis. Only children with at least 3 watery or liquid stools, with or without vomiting were included in the study. The minimum sample size (118) was determined using the Lorentz formula with a prevalence equal to 0.08 [17].

The retrospective study focused on hospital data from January 2016 to December 2018 from two health institutions: HUME and HNDA. These data included the results of the stool culture and direct microscopy examinations for children aged 5 and below, hospitalized or seen for acute non-bloody gastroenteritis. Although RVA was not investigated, triangulating the results of the 1st and 2nd parts make it possible to determine the possible contribution of RVA in the aetiology of childhood gastroenteritis within these three years (Table 1), and thus use this to infer the general prevalence of RVA in N’Djamena.

**Table 1:** Distribution of the number of samples per hospital in the two studies

**Study type**	Hospitals				
	CHUME	HNDA	HATC	CHUBS	Total
Cross-sectional	56	45	24	16	141
Retrospective	1250	1342	0	0	2592

### The cross-sectional study

Using a questionnaire, socio-demographic data and clinical parameters of each participating patient were collected. As well, approximately 5 ml or 5 mg of faeces samples from each patient were collected in a sterile stool container, labelled and transported in a cooler (4–8° C) to the laboratory *(Laboratoire de Virology, Institut de Recherche en Elévage pour le Développement” (IRED))*. From each sample, 10% suspension using Phosphate Buffered Saline as diluent was prepared. Each suspension was subjected to VP6 antigen detection by sandwich ELISA (Human Rotavirus antigen, RVA Ag ELISA Kit, Sunlong Biotch). According to manufacturer’s instructions, Optical Density (OD) readings value equal to or above the cut-off value (0,2384) was considered positive and OD value below cut-off was considered negative [18]. The assay was considered valid when the mean value of the positive control ≥ 1.00 and the mean value of the negative control ≤ 0.10 [18].

To determine the Vesikari scores of the patients for gastroenteritis severity the following clinical parameters were used : maximum number of stools per day and duration of diarrhoea, maximum number of vomiting per day and duration of vomiting, temperature, dehydration and treatment as described by the Vesikari Clinical Severity Scoring System Parameters and Scores [19]. Total score obtained was used to classify the severity level of gastroenteritis as mild, moderate, and severe for Vesikari scores below 7, between 7 and 10 and above 11, respectively [19].

### Statistical analysis

Statistical analyses were performed using SPSS 23 software (IBM SPSS Statistics 23.0). Statistical distributions were compared using Chi-square test with 95% confidence interval and a p-value of 5%. The distribution of Vesikari clinical severity score was compared between children who tested positive for rotavirus versus those tested negative. The Vesikari clinical severity score was grouped into mild, moderate and severe.

## Results

### Cross-sectional study

#### Overview of gastroenteritis

A total number of 141 stool samples were collected. The results are summarized in Table 1. The mean age of gastroenteritis affected children was 17.12 months with a male predominance of 59% (83/141). Two cases of death, all males were recorded of which one case was an 18-monthold child, who had persistent diarrhoea and dehydration, and one case known to be HIV infected.

#### Prevalence and severity of RVA infection

RVA infection was detected in 12.76% (18/141) cases, and 61.11% (11/18) of which were male. The most affected age group was that of 0 to 11 months with 44.4% (8/18). HNDA hospital had 44.4% (8/18) of the cases, followed by CHUME hospital with 33.3% (6/18) (Table 2). Among the positive cases, one case of death of a child suffering from gastroenteritis was noted.

**Table 2:** Frequency of RVA gastroenteritis according to the sex, age, hospitals, nutritional status, breastfeeding, and weaning (n = 18)

	Positive RVA n (%)	Negative RVA n (%)	*p-value*
Sex			
Male	11 (61.1)	72 (58.5)	0.83
Female	7 (38.9)	51 (41.5)	
Age (month)			
0–11	8 (44.4)	48 (39.0)	
13–23	5 (27.8)	42 (34.2)	0.03
24–59	5 (27.8)	33 (26.8)	
Hospitals			
CHUME	6 (33.3)	50 (40.6)	
CHUBS	1 (5.6)	15 (12.2)	0.27
HATC	3 (16.7)	21 (17.1)	
HNDA	8 (44.4)	37 (30.1)	
Nutritional status			
Normal	4 (22.2)	55 (44.7)	
Moderate	6 (33.3)	48 (39.0)	0.004
Severe	8 (44.5)	20 (16.3)	
Exclusive breastfeeding			
Yes	0 (0)	19 (21.8)	0.14
No	8 (100)	68 (78.2)	
Weaning			
Before 2 years	5 (50.0)	11 (30.6)	0.26
For 2 years	5 (50.0)	25 (69.4)	

#### Risk factors

Among the 18 RVA positive cases, 22.2% (4/18) had normal nutritional status, 33.3% (6/18) moderate acute malnutrition, and 44.5% (8/18) had severe acute malnutrition.

For the feeding mode, no child under exclusive breastfeeding was found among the infected. Concerning early weaned children, half (50%) were infected by RVA (Table 2). Among children with rotavirus infection, 22.2% have diarrhoea lasting longer than six days and 38.9% of these diarrhoeas occur more than six times a day. Children with severe dehydration represent 44.5% (Table 3). The mean Vesikari score shows that 38.8% of children have a high level of severity and 41.1% have a moderate level (Table 3).

**Table 3:** Distribution of cases within the clinical parameters of the Vesikari scoring for severe gastroenteritis (n = 18).

Risk factor	n (%)
Diarrhoea Duration (days)	
1–4	8 (44.4)
5	6 (33.3)
≥ 6	4 (22.2)
Frequency of diarrhoea per day	
3	1 (5.5)
4–5	10 (55.6)
≥ 6	7 (38.9)
Duration of vomiting (days)	
0	6 (33.3)
1	2 (11.1)
2	5 (27.8)
≥ 3	5 (27.8)
Frequency of vomiting per day	
0	6 (33.3)
1	1 (5.6)
2–4	6 (33.3)
≥ 5	5 (27.8)
Temperature (°C)	
37.1–38.4	3 (20)
38.5–38.9	10 (66.7)
≥ 39.0	2 (13.3)
Dehydration status	
None	4 (22.2)
Some dehydration	6 (33.3)
Severe dehydration/shock	8 (44.5)
Treatment	
Rehydrated	6 (33.3)
Admitted	12 (66.7)
Severity Category	
Mild	19 (17.1)
Moderate	49 (44.1)
Severe	43 (38.8)

### Retrospective study

In total, 2,592 diarrheic gastroenteritis cases were recorded with 980 (37.80%) cases due to diarrheagenic pathogens listed above. We found that the male population has a slightly higher incidence with 54.5% (n = 534) and a sex ratio of 1.37. The most affected age group was children aged between 0 and 11 months (57.3%) (Table 4).

**Table 4:** Frequency of gastroenteritis according to the sex, age and hospitals between 2016 and 2018

Risk Factor	Positive test *n* (%)	Negative test *n* (%)
Tests		
Stool ova and parasite	901 (38.4)	1442 (61,6)
Stool culture	79 (31.7)	170 (68.3)
General frequency	980 (37.8)	1612 (62.2)
Sex		
Male	534 (54.5)	966 (59.9)
Female	446 (45.5)	646 (40.1)
Age (month)		
0–11	562 (57.3)	508 (51.8)
12–23	282 (28.8)	610 (62.2)
24–59	136 (13.9)	494 (50.4)
Hospitals		
CHUME	472 (48.2)	778 (48.3)
HNDA	508 (51.8)	834 (51.7)

**Fig. 1:**
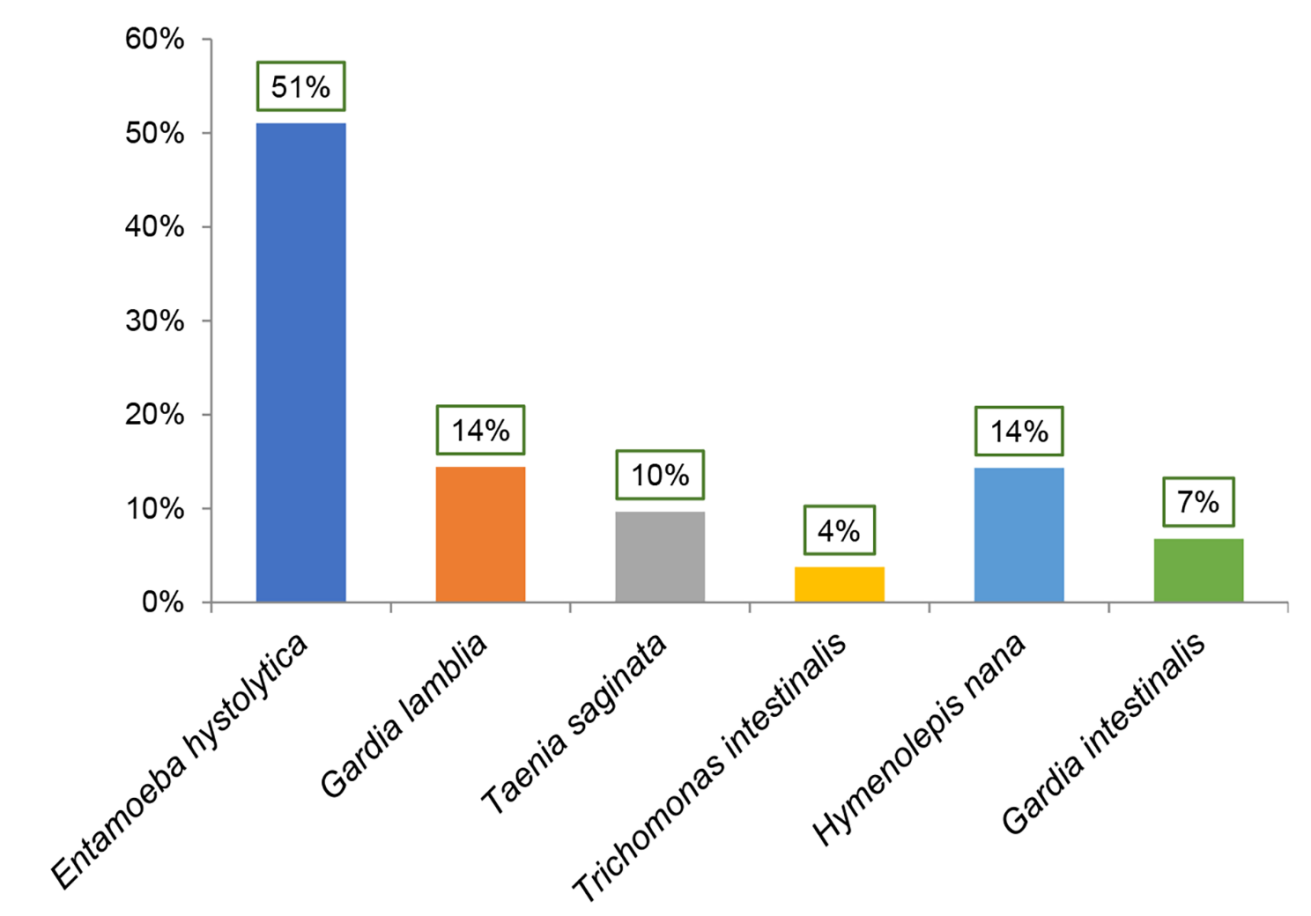
Parasites identified in the stool ova and parasites test between January 2016 and December 2018

**Fig. 2:**
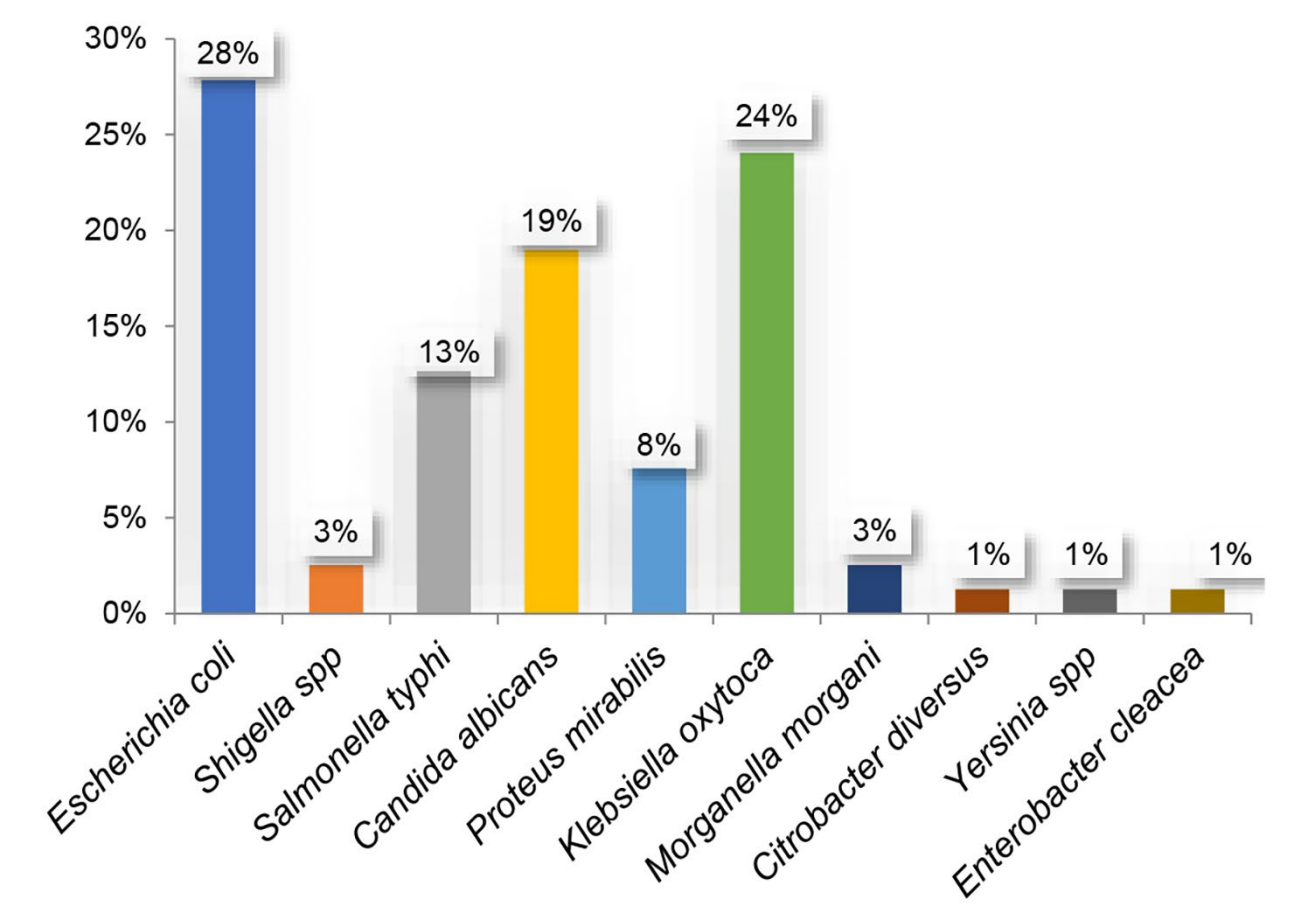
Microorganisms identified in stool culture between January 2016 and December 2018 *Emtamoeba hystolitica, Gardia lamblia, Taenia saginata, Trichomonas intestinalis, Hymenolepis nana, Gardia intestinalis, Escherichia coli, Shigella spp, Salmonella typhi, Candida albicans, Proteus mirabilis, Klebsiella oxytoca, Morganella morgani, Citrobacter diversus, Yersinia spp, Enterobacter cleacea*

Among the parasites sought, *Entamoeba histolytica* (51%) followed by *Gardia lamblia* (14%) and *Hymenolepus nana* (14%) predominated in the stool ova and parasites test (Fig. 1) while for stool culture, *Escherichia coli* (28%), *Klebsiella oxytoca* (24%), *Candida albicans* (19%) and *Salmonella typhi* (13%) were the most frequently identified (Fig. 2).

**Fig. 3:**
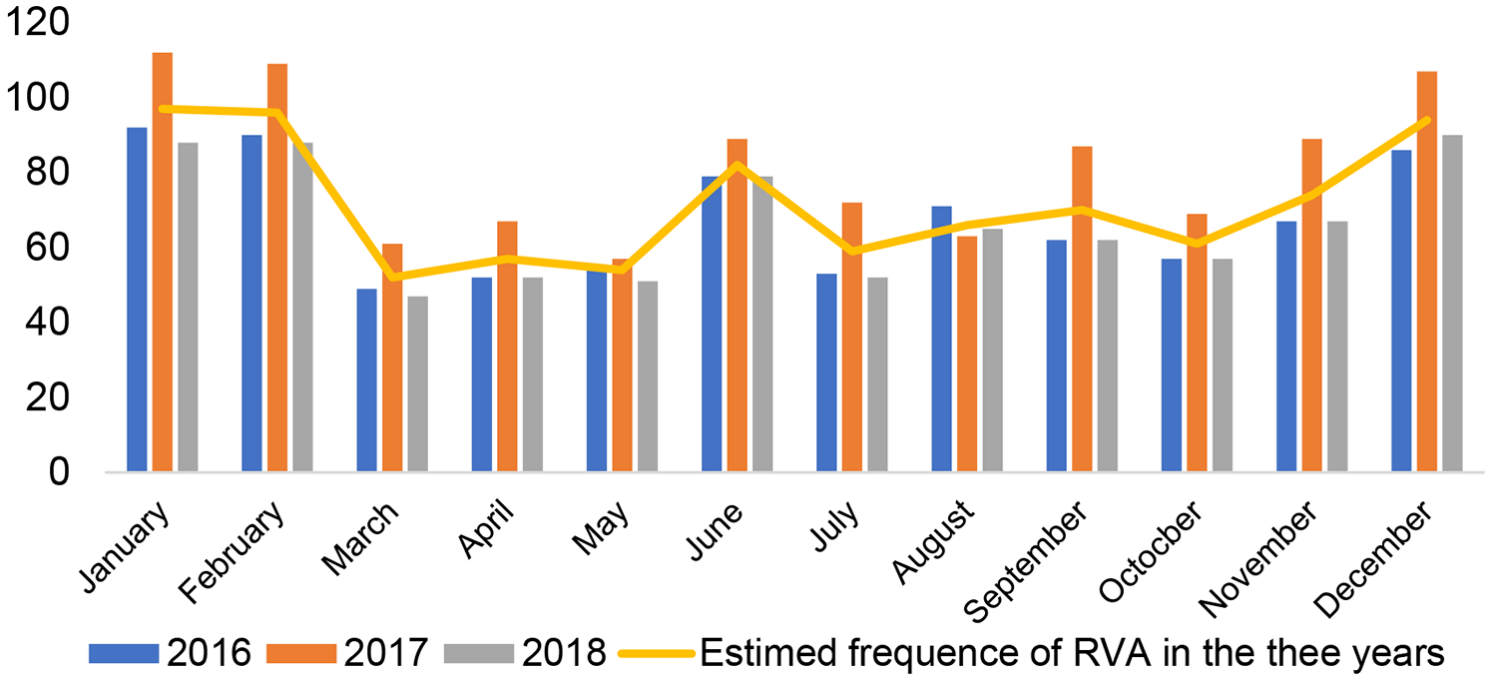
Seasonal frequency of diarrhoea and estimated frequency of RVA between 2016 and 2018

Figure 3 shows the prevalence of diarrheal infections, and that of rotaviruses. As noted the pic for both diarrhoea and positive RVA, are high between December and February. This period in our study area corresponds to that of the dry season.

## Discussion

The implication of RVA in childhood gastroenteritis was evidenced among children under five in N’Djamena. Like in many Sub-Sahara African countries, RVA infection represents a serious public health burden in Chad republic. This burden goes un-noticed, because, RVA is not taken into account in the laboratory diagnosis processes of gastroenteritis, and there currently no RVA surveillance in Chad republic. The present study serves as indicator of the fact that RVA is a major cause of childhood diarrhoea, and may as well serve as a reference useful in the initiation of RVA surveillance in Chad. This is the first study of its kind, focussing only on RVA infection in the targeted population in Chad Republic.

Previous investigations carried out in sub-Sahara Africa highlighted the implication of RVA in childhood gastroenteritis with 30 to 39% mortality rate [3], but, with evidence of 50% or more reduction from the pre-vaccination to post-vaccination period [14] with accompanying reduction in the mortality rate [3]. Chad ministry of public health did not yet integrate RVA vaccination in the national immunization program. The results of the present study, although carried in urban area and in well-equipped hospitals showed a prevalence of 12.76% in the cross sectional study, and thus indicative that RVA is endemic in Chad republic. This apparently low prevalence is close to the prevalence obtained by Muendo in 2018 in Kenya (14.5%) [20] in a post-vaccination investigation, should not be used to minimize RVA burden in Chad Republic. This is close to the proportion obtained in Chad in 2016 by Bruno, which was 8%. Because the present study did not include, rural areas and it focussed only on one of the most developed part of the country where hygienic conditions are acceptable. This view is comforted by a recent study carried out in the University Hospital Center of Mother and Child, N’Djamena, showed rotavirus prevalence of 48.24% [16], a figure, much more in conformity with RVA pre-vaccine prevalence in many African countries. Another reason that can justify the low RVA prevalence is that we carried out this cross sectional study during the rainy season, a period of the year not favourable to long settlement of pathogens on surfaces. In a recent study carried out in the Littoral region of Cameroon, a neighbouring country to Chad republic, Ghapoutsa et al. obtained a RVA prevalence of 54.6% using the same method (VP6 protein detection by ELISA, 57% of which were fully vaccinated [13]. Giving the high flow of goods, cattle and humans between Chad and Cameroon, this study could be greatly suggestive to the heath authority in Chad, not only to introduce RVA vaccination, but also, to engage prior study to detect circulating strains for a better choice of the vaccine to be applied.

As observed with previous studies [13, 14, 21, 22] that younger children (0–12 months of age) are more susceptible to RVA than older ones. In the present study RVA infection within the age group of 0–11 months has the highest prevalence rate (44.4%, *p = 0.03*) (Table 2). This is attributable to the immature immune system [23], especially in the context where exclusive breastfeeding [11]is not applied. Exclusive or intensive breastfeeding at younger age provides protection through the mother secretory IgA [24]. But, as observed, none of the affected child was on exclusive breastfeeding, even though it is expected that this age bracket should be the one under intensive breastfeeding. This is further supported by the finding that none of the children under exclusive breastfeeding was found infected in the present study.

Among the positive cases, the prevalence was higher in boys (61.1%) than in girls (38.9%), though with no statistical significance (*p = 0.83*) (Table 2). Male children develop vigorous physical features compare to female children, and this is thanks to the involvement of sex hormones. The impact of sex hormones on cytokine balance and on the T-helper 1/T-helper 2 has been shown to reduce resistance to infectious disease in male children compared to female children [25]. The RVA infection prevalence obtained in the present study is close to the 66.67% and 51.9% of males obtained in a similar studies respectively in Cameroon [10] and the Democratic Republic of Congo with a high proportion for the female sex(51,9%) [26].

Based on the hospital site, more cases of RVA gastroenteritis were obtained in HNDA and CHUME with respectively 44.4% and 33.3% (*p = 0.27*) (Table 2). This high prevalence could be justified by the fact that these two hospitals have the highest number of children sampled in the study. These two hospitals also have Nutritional and Therapeutic Units and thus stand as reference hospitals for children.

Nutritional status has a great impact on development of the immune system; 44.5% of the infected children had severe acute malnutrition and 33.3% moderate acute malnutrition. This make malnutrition the highest (5%, *p = 0.04*) (Table 2) risk factor of RVA infection, and thus rank RVA gastroenteritis a poverty related disease, and also justifies why RVA is endemic and highly prevalent in low income resource countries.

The Vesikari Clinical Severity score among rotavirus infected children obtained shows 44.1% were rated moderate, 38.8% rated severe and 17.1% rated mild. This shows the severe level of rotavirus infection in these children with a fairly low nutritional status obtained. This can be explained by the young age of infected children 0–11 months (44.4%) exposing them more easily to the risks of severity. According to the World Health Organization scientific working group, most cases of rotavirus infections occur in children between 6 and 24 months with a peak incidence at 9 to 12 months [27].It is postulated that younger children tend to be at an increased risk of developing severe dehydration due to their small body size, as they lose a greater portion of their total fluid volume during the illness [28].

For the retrospective study, only 37.8% of stool examinations at the CHUME and HNDA were positive, but all the children admitted presented with gastroenteritis. This striking low positive diagnosis (compared to 62.20% negative stool examination) may be justified by the failure to consider seeking for viral infections during laboratory diagnosis. Yet, the high implication of RVA in childhood gastroenteritis has been greatly propagated by WHO.RVA is considered the leading cause of death associated with childhood gastroenteritis worldwide [3], and the Chad republic health authorities should engage to setting up facilities empowered with RVA detection capacity.

Among the microorganisms identified in stool, *Entamoeba histolytica* predominated with 51% in the stool ova and parasites test (Fig. 2) and *Escherichia coli* predominated with 28% for stool culture (Fig. 1). This could be explained by the fact that *Entamoeba histolytica* and *Escherichia coli* are ubiquitous microorganisms that can cause more impact in children under five (5) years old. However, considering the panel of pathogens targeted by the diagnostic methods used locally, only 62.2% of cases diarrhoea has a known cause. It is thus evident that 37.8% constituting the remaining cases are due to other pathogens not included in the diagnostic panel. Among these, RVA is surely a prime cause, very likely with co-infections, as recently noticed in the littoral region of Cameroon [13].

About the other pediatric enteroviral infections, the rotavirus prevalence as revealed in the cross-sectional study is suggestive that other diarrheagenic virus are associated with diarrhea in children N’Djamena.

Season also affect infectious disease prevalence, particularly that of RVA gastroenteritis in children. Indeed, given the non-consideration of the search for rotavirus among the pathogen panel in these children, a significance threshold was determined with p-value = 0.1%< 5%. RVA prevalence was found to be higher between December and march corresponding to the cold period of the dry season than in the rainy season (Fig. 3). This finding may result from the abundance of dust transported by the wind during this period to contaminate surfaces, favouring the infection, especially in poor hygienic environments as shown by the results of Gonzalez-Martin regarding the presence of rotaviruses in dust [29]. This result could also be explained not only by the transmission favored by the high excretion of rotavirus in the stools but also by the resistance and the low infectious dose of rotaviruses. Given that among the children included in the study, some were in nutritional and therapeutic units, contamination of surfaces, gowns, children’s objects or toys favored nosocomial viral dissemination [30, 31].This observation is similar to those of Patel in 2013 [32] and Tsolenyanu in 2017 [33], although a different observation was made by Bruno et al. [17] and Godfrey et al. [14] who noticed high frequencies in the rainy season.

The results obtained in two aspects of the present study, 18/141 RVA cases for the cross-sectional part, and 980/2592 proven infectious diarrhoea cases for the retrospective part, may be used to estimate the contribution of RVA in the study site to 343 cases out of a total of 2592 diarrhoea cases within the period of January 2016 to December 2018. If we use this prevalence and considering the total case of diarrhea in the retrospective and the prospective study, the total burden of RVA in N’Djamena within the tree years is 349.

### Limitations

The major difficulty encountered was the missing data from hospitals where we did the study. This led to the exclusion of many cases that could have improve our results in both parts of the study. 

## Conclusion

RVA is endemic in Chad Republic with an infection prevalence of at least 12.76%. From this study, we estimate that the total number of children affected by RVA could be 343 within the period of the retrospective study. These results should prompt the health authorities of the country to initiate studies in view of RVA vaccination, and further studies to determine dominant circulating strains as well as the determination of strains of zoonotic origin in circulation.

## Data Availability

The datasets used and analysed during the current study are available from the corresponding author.

## List of Abbreviations

CHUBS: *Centre Hospitalo-Universitaire le Bon Samaritain*
CHUME: *Centre Hospitalier Universitaire de la Mère et de l’Enfant*
ELISA: Enzyme Linked ImmunoSorbent Assay
HATC: *Hôpital de l’Amitié Tchad-Chine*
HNDA: *Hôpital Notre Dame des Apôtres*
IRED: *Institut de Recherche en Elévage pour le Développement*
RV: Rotavirus
RVA: Group A Rotavirus
VP6: Viral Protein 6

## References

[1] Bishop R, Davidson G, Holmes I et, Ruck B. «Virus particles in epithelial cells of duodenal mucosa from children with acute nonbacterial gastroenteritis,» *Lancet*, vol. 2, n° %11281-3, 1973. 10.1016/s0140-6736(73)92867-5 4127639

[2] Estes M. «Rotaviruses and their replication,» *Knipe DM, Howley PM, eds. Fields virology*, Vols. %1 sur %2vol 2, 4th edn., n° %11747–86., 2001.

[3] Troeger C, Ibrahim A et, Beth D. «Rotavirus Vaccination and the global burden of Rotavirus Diarrhea among Children younger than 5 years,» JAMA Pediatr, 2018. 10.1001/jamapediatrics.2018.1960 PMC6233802 30105384

[4] Global Burden of Disease, «Causes of Death Collaborators. Global, regional, and national age-sex specific mortality for 264 causes of death, 1980–2016: asystematic analysis for the Global Burden of Disease Study 2016.,» *Lancet*, 2016. 10.1016/S0140-6736(17)32152-9 PMC5605883 28919116

[5] Kotloff K, Nataro J, Blackwelder W, Nasrin D, Farag T et, Myron M. Burden and a etiology of diarrhoeal disease in infants and young children in developing countries (the Global Enteric Multicenter Study, GEMS): a prospective, case-control study., 2013. 10.1016/S0140-6736(13)60844-2 23680352

[6] WHO., «World health statistics,» 2013. [En ligne]. Available:. http://www.who.int/gho/publications/world_health_statistics/2013/en/. [Accès le 22 june 2019].

[7] WHO., «Weekly epidemiological record,» 2007.

[8] Aballéa S, Aurélie M, Sibilia Q, Stuart C, Stavros P, et Mondher T. «A review of the critical literature on the economic evaluations of Rotavirus vaccination by health,» *Journal Human Vaccines & Immunotherapeutic*, Vols. %1 sur %2Volume 9 - Issue 6., 2013. 10.4161/hv.24253 PMC3901817 23571226

[9] Chen S, Tan L, Huang L et, Chen K. «Rotavirus infection and the current status of rotavirus vaccines,» J Formos Med Association n° %1111(4), pp. pp.183–93, 2012. 10.1016/j.jfma.2011.09.024 22526206

[10] Eteme FL, Charles F, Fernand T, Désiré N, Angeline B, Ndze V, Grace k, Estella T et, Donatien G. «Molecular epidemiology of group a rotavirus associated with gastroenteritis in children under 5 in the city of Yaoundé (Cameroon),» J Biology Chem Sci, 2015.

[11] Morrow A, Ruiz-Palacios G, Altaye M, Jiang X, Guerrero M, Meinzen-Derr J, Farkas T, Chaturvedi P. L. Pickering et D. Newburg, «Human milk oligosaccharides are associated with protection against diarrhea in breast-fed infants,» J Pediatr, 2004. 10.1016/j.jpeds.2004.04.054 15343178

[12] Waggie Z, Hawkridge A et, Hussey GD. «Review of rotavirus studies in Africa: 1976–2006,» *Journal of Infectious Diseases*, pp. pp.S23-S33, 2010. 10.1086/653554 20684708

[13] Ghapoutsa RN, Boda M, Gautam R et, Ndze VN. «Detection of diarrhoea associated rotavirus and co-infection with diarrhoeagenic pathogens in the Littoral region of Cameroon using ELISA, RT-PCR,» 2021. 10.1186/s12879-021-06318-x PMC8237514 34182936

[14] Godfrey O, Zhang W, Amponsem-Boateng C, Bonney Oppong T, Zhao Q et, LI D. «Evidence of rotavirus vaccine impact in sub-saharan Africa: systematic review and meta-analysis,» *PLoS one*, n° %115(4) p.e0232113, 2020. 10.1371/journal.pone.0232113 PMC7185587 32339187

[15] Todd S, Page NA, Steele AD, Peenze I et, Cunliffe NA. «Rotavirus strain types circulating in Africa: review of studies published during 1997–2006,» *Journal of Infectious Diseases*, pp. pp.S34-S42, 2010. 10.1086/653555 20684715

[16] Fombotioh N, Abakar IL, Nadjioroum N-A, Mouktar AA. B. Boy Otchom et H. Mahamat Alio, «The Prevalence of Rotavirus and Adenovirus in Children 0–5 years,» *International Journal of Scientific Advances*, vol. 2, n° %1ISSN: 2708–7972, 2021.

[17] Bruno A, Harouna S, Ali O, Frida B, Nafissa D, Ahmat O, Susan S et, Abdelkerim T. «Diagnosis by qualitative molecular biology can improve the management of severely acutely malnourished children suffering from diarrhea: case of TUN from the Chad-China Friendship Hospital. N’Djaména, ; 2016. Chad.,».

[18] Sunlong. «Human Rotavirus IgA(RV-IgA)ELISA Kit,» 2019. [En ligne]. Available: https://sunlongbiotech.com/goods.php?id=28566.

[19] Ruuska T et, Vesikari T. «Rotavirus disease in finnish children: use of numerical scores for clinical severity of diarrhoeal episodes,» Scand J Infect Dis, 1990. 10.3109/00365549009027046 2371542

[20] Muendo C, Ahmed L, Rashmi k, Boniface O, Egondi T et, Pamela N. «Prevalence of rotavirus infection among children with acute diarrhoea after rotavirus vaccine introduction in Kenya, a hospital cross-sectional,» BMC Pediatr, 2018. 10.1186/s12887-018-1291-8 PMC6180366 30309343

[21] Tate J, Burton A, Boschi-Pinto C. et U. Parashar, «Global, regional and national estimates of rotavirus mortality in children under 5 years old between 2000 and 2013.,» 2016. 10.1093/cid/civ1013 PMC11979873 27059362

[22] Esona MD, Leanne WM, Mary WE, Slavica RM, Rashi G, Charity P, Rangaraj S, Christopher JH, Julie BA, Janet EA, Eileen KJ, Mary AS, Monica MM, Natasha H, James C, Geoffrey WA, Daniel PC. P. D. Umesh et B. D. Michael, «Rotavirus Genotype Trends and Gastrointestinal Pathogen Detection in the United States, 2014–2016: Results From the New Vaccine Surveillance Network,» *The Journal of Infectious Diseases*, Vols. %1 sur %2224 (9):1539–1549, n° %110.1093/infdis/jiab177, 2021. 10.1093/infdis/jiab177 33822119

[23] WHO Scientific Working., «Rotavirus and other viral diarrhoeas: WHO scientific working,» 1980. PMC2395787 6249509

[24] Blutt SE et, Conner ME. «The gastrointestinal frontier: IgA and viruses,» *Front Immunol*, vol. 4:402, n° %110.3389/fimmu.2013.00402, 2013. 10.3389/fimmu.2013.00402 PMC3842584 24348474

[25] Muenchhoff M et, Goulder PJ. «Sex differences in pediatric infectious diseases,» *J infect Dis*, Vols. %1 sur %2S120-6, n° %110.1093/infdis/jiu232, 2014. 10.1093/infdis/jiu232 PMC4072001 24966192

[26] Sangaji M, Olivier M, Augustin M, Oscar N. Etude épidémio-clinique des diarrhées aiguës à Rotavirus chez les nourrissons, à l’hôpital Jason Sendwe de Lubumbashi, République Démocratique du Congo, Pan Afr Med J, vol. 21, no. 113, 2015. 10.11604/pamj.2015.21.113.5737 PMC4546712 26327950

[27] WHO., «Weekly Epidemiological Record,» 1999.

[28] Guarino A, Ashkenazi S, Gendrel D, Lo Vecchio A, Shamir R, Szajewska H (2014). «European society for pediatric gastroenterology, hepatology, and nutrition/european society for pediatric infectious diseases evidence-based guidelines: update,». J Pediatr Gastroenterol Nutr.

[29] Gonzalez-Martin C, Coronado-Alvarez N, Teigell-Perez N, Diaz-Solano R, Exposito FJ, Diaz JP. D. W. Griffin et B. Valladares, «Analysis of the Impact of African Dust Storms on the Presence of Enteric Viruses,» n° %11680 – 8584 print / 2071 – 1409, 2018.

[30] Gianino P, Mastretta E, Longo P, Laccisaglia A, Sartore M, Russo R, Maz A et, Mazzaccara A. «Incidence of nosocomial rotavirus infections, symptomatic and asymptomatic, in breast-fed and non-breast-fed infants,». J Hosp Infect n° %1doi. 2003;13–7. 10.1053/jhin.2001.1129. 10.1053/jhin.2001.1129 11825046

[31] Herruzo R, Omeñaca F, García S, Diez J. et A. Sánchez-Fauquier, «Identification of risk factors associated with nosocomial infection by rotavirus P4G2, in a neonatal unit of a tertiary-care hospital,» *Clinical Microbiology and Infection*, 10.1111/j.1469-0691.2008.02667.x 10.1111/j.1469-0691.2008.02667.x 19210698

[32] Patel M, Pitzer V, Alonso W, Tate J, et Pothier P. «Rotaviruses and their prevention.,» *Therapeutic medicine / pediatrics*, n° %1150: 23–27, 2013.

[33] Tsolenyanu E, Fiawoo M, Akolly D, Guedenon K et, Dangra A. Extent of Rotavirus gastroenteritis in children under five, vol. 9, 2017.

